# Errata

**DOI:** 10.36660/abc.20220302

**Published:** 2022-05-04

**Edição de Dezembro de 2021, vol. 117(6), págs. 1093-1103**

No Artigo Original “O Efeito Anti-Hipertensivo de Sauromatum Guttatum Mediado por Efeitos Vasorrelaxante e Depressivos Miocárdicos“, com número de DOI: https://doi.org/10.36660/abc.20200055, publicado no periódico Arquivos Brasileiros de Cardiologia, 117(6):1093-1103, na página 1093, corrigir o nome da autora Rabia Bibi para Bibi Rabia.

**Edição de Fevereiro de 2022, vol. 118(2), págs. 525-529**

Na Carta Científica “Uma Apresentação Rara de COVID-19 com Embolia Pulmonar“, com número de DOI: https://doi.org/10.36660/abc.20210350, publicado no periódico Arquivos Brasileiros de Cardiologia, 118(2):525-529, na página 525, corrigir o nome Özgenur Günçkan para Özgenur Güçkan.

**Edição de Fevereiro de 2022, vol. 118 (2), págs. 536-547**

Na “Atualização da Diretriz de Avaliação Cardiovascular Perioperatória da Sociedade Brasileira de Cardiologia: Foco em Manejo dos Pacientes com Intervenção Coronária Percutânea – 2022”, com número de DOI: https://doi.org/10.36660/abc.20220039, publicado no periódico Arquivos Brasileiros de Cardiologia, 118(2): 536-547, na página 543 da versão em português, figura 1, corrigir o texto do quadrado laranja de “30 dias a < 6 meses” para “30 dias a < 3 meses”. A figura 1 está correta na versão inglês.

