# Supplementary material for: Errata
Source: Arq Bras Cardiol. 2022 May 4;118(5):1005. [Article in Portuguese] doi: 10.36660/abc.20220302 (PMC9368880; doi:10.36660/abc.20220302)
Supplement: Translation

# Translation: Erratum

**December 2021 Issue, vol. 117(6), pages 1093-1103**

In the Original Article “Antihypertensive Activity of Sauromatum guttatum Mediated by Vasorelaxation and Myocardial Depressant Effects”, with DOI: https://doi.org/10.36660/abc.20200055, published in the journal Arquivos Brasileiros de Cardiologia, 117(6):1093-1103, in page 1093, correct the author’s name Rabia Bibi to Bibi Rabia.

**February 2022 Issue, vol. 118(2), pages 525-529**

In the Research Letter “A Rare Presentation of COVID-19 with Pulmonary Embolism”, with DOI: https://doi.org/10.36660/abc.20210350, published in the journal Arquivos Brasileiros de Cardiologia, 118(2):525-529, in page 525, correct the author’s name Özgenur Günçkan to Özgenur Güçkan.

**February 2021 Issue, vol. 118 (2), pages 536-547**

In the “Update of the Brazilian Society of Cardiology’s Perioperative Cardiovascular Assessment Guideline: Focus on Managing Patients with Percutaneous Coronary Intervention – 2022”, with DOI number: https://doi.org/10.36660/abc.20220039, published in the journal Arquivos Brasileiros de Cardiologia, 118(2): 536-547, on page 543 of the Portuguese version, Figure 1, correct the text of the orange square from “30 dias a < 6 meses” to “30 dias a < 3 meses”. Figure 1 is correct in the English version.
